# Expression of a Cytochrome P450 Gene from Bermuda Grass *Cynodon dactylon* in Soybean Confers Tolerance to Multiple Herbicides

**DOI:** 10.3390/plants11070949

**Published:** 2022-03-31

**Authors:** Ting Zheng, Xiaoxing Yu, Yongzheng Sun, Qing Zhang, Xianwen Zhang, Mengzhen Tang, Chaoyang Lin, Zhicheng Shen

**Affiliations:** 1 State Key Laboratory of Rice Biology, Institute of Insect Sciences, College of Agriculture and Biotechnology, Zhejiang University, Hangzhou 310000, China; lehaax@zju.edu.cn (T.Z.); 11616071@zju.edu.cn (X.Y.); zsyz@zju.edu.cn (Y.S.); chylin@zju.edu.cn (C.L.); 2 Hangzhou Ruifeng Biosciences Co., Ltd., 1500 Wenyi Road, Building 1, Room 103, Hangzhou 310000, China; zhangqing@rfgene.cn (Q.Z.); tangmengzhen@rfgene.cn (M.T.); 3 Agricultural Experiment Station, Zhejiang University, Hangzhou 310000, China; bestzxw@163.com

**Keywords:** *Cynodon dactylon*, P450, herbicide, transgenic

## Abstract

Bermuda grass (*Cynodon dactylon*) is notoriously difficult to control with some commonly used herbicides. We cloned a cytochrome P450 gene from Bermuda grass, named *P450-N-Z1*, which was found to confer tolerance to multiple herbicides in transgenic *Arabidopsis*. These herbicides include: (1) acetolactate synthase (ALS) inhibitor herbicides nicosulfuron and penoxsulam; (2) p-hydroxyphenylpyruvate dioxygenase (HPPD)-inhibiting herbicide mesotrione; (3) synthetic auxin herbicide dicamba; (4) photosynthesis inhibitor bentazon. We further generated transgenic soybean plants expressing *P450-N-Z1*, and found that these transgenic soybean plants gained robust tolerance to nicosulfuron, flazasulfuron, and 2,4-dichlorophenoxyacetic acid (2,4-D) in greenhouse assays. A field trial demonstrated that transgenic soybean is tolerant to flazasulfuron and 2,4-D at 4-fold and 2-fold the recommended rates, respectively. Furthermore, we also demonstrated that flazasulfuron and dicamba are much more rapidly degraded in vivo in the transgenic soybean than in non-transgenic soybean. Therefore, *P450-N-Z1* may be utilized for engineering transgenic crops for herbicide tolerance.

## 1. Introduction

Genetically modified crops for glyphosate tolerance have been widely planted commercially, and have delivered significant economic benefits such as increasing productivity and efficiency in farm management [1]. However, the evolution of glyphosate resistance in weeds due to its prolonged intensive use has become a serious threat to the efficient weed management system of glyphosate-tolerant crops. Fifty-five weed species have been reported to have developed glyphosate resistance by February 2022 and the number of resistant weed species continues to increase every year [2,3].

To manage herbicide resistance development in weeds, an effective practice is to use herbicides with different modes of action (MOA), either in rotation or in combination as mixtures [4]. Transgenic crops resistant to dicamba and 2,4-dichlorophenoxyacetic acid (2,4-D) have been developed to control the glyphosate-tolerant weeds [5,6]. However, both dicamba and 2,4-D are effective only for control of broadleaf weeds but not monocot weeds. Furthermore, the resistance to these two herbicides in weeds has also been reported due to their long history of field applications [7,8,9]. Therefore, development of transgenic crops resistant to more herbicides with different modes of action is still highly desirable to enhance and improve the current weed control technologies.

Plants have a large family of cytochrome P450 (P450s) genes. In addition to their critical roles in the synthesis of lignin, pigment, defending compound, fatty acid, hormone, and signaling molecules, a number of plant P450s are vital for the metabolism and detoxification of herbicides [10,11]. P450s metabolize herbicides mainly by alkyl-hydroxylation, N-demethylation, O-demethylation, and aryl-hydroxylation [12]. Cytochrome P450s from *Helianthus tuberosus* [13,14], *Nicotiana tabacum* [15], *Arabidopsis thaliana* [16], *Glycine max* [17,18], *Gossypium hirsutum* [19], *Triticum aestivum* [20], *Oryza sativa* [21,22,23], *Zea mays* [24,25,26], *Echinochloa phyllopogon* [27,28], and *Loilium rigidum* [29] have been studied for their involvement in the metabolism of various herbicides. However, these plant P450 genes have not yet been utilized to develop commercial transgenic herbicide-tolerant crops, likely due to their relative low efficacy or the unfavorable competitiveness of their target herbicides.

Bermuda grass, *Cynodon dactylon* (L.) Pers, is notoriously tolerant to quite a few herbicides [30,31,32]. We hypothesized that this plant may harbor a robust cytochrome P450 gene for detoxification of various herbicides. Such herbicide detoxification genes may be preferred for engineering crop tolerance to border spectrum herbicides. Here, we report the cloning of a cytochrome P450 gene that confers tolerance to multiple herbicides, and the creation of transgenic herbicide-tolerant soybean by expressing this gene.

## 2. Results

### 2.1. Cloning and Characterization of a Cytochrome P450 Gene from Bermuda Grass

To clone the cytochrome P450 gene responsible for the herbicide tolerance in Bermuda grass, the conservative region of the known cytochrome P450 genes from other plant species involved in herbicide tolerance was identified. A pair of primers was designed based on this conservative region to amplify a cDNA fragment by RT-PCR. Two different but highly similar partial cDNA fragments were obtained, and subsequently their full-length cDNA was cloned by the rapid amplification of cDNA ends (RACE) method. The two full-length cDNAs encode two polypeptides of 517 and 531 amino acid residues, named as P450-N-Z1 and P450-N-Z2, respectively (GenBank accessions: KT184658.1 and KT184659.1). 

**Figure 1:**
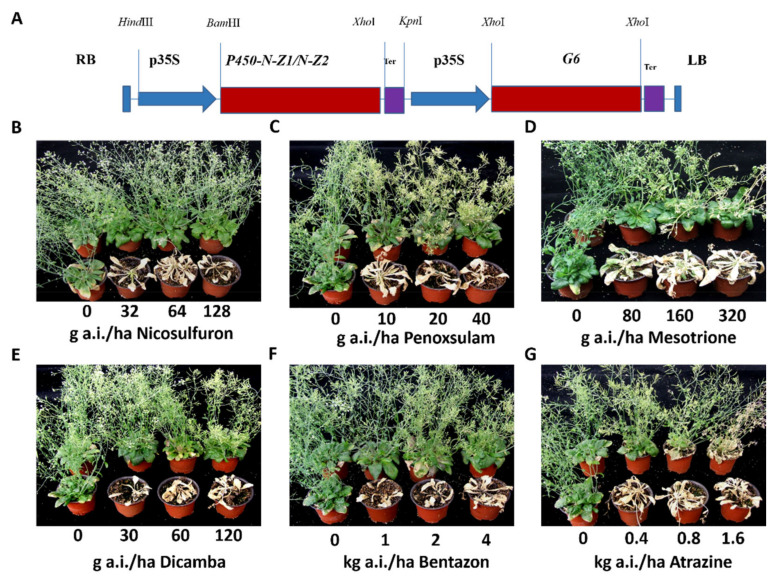
Herbicide tolerance assays of *Arabidopsis* transformed with T-DNA with *P450-N-Z1* expression cassette. (**A**) The diagram of the T-DNA for *Arabidopsis* transformation. (**B**) *P450-N-Z1* transgenic *Arabidopsis* challenged with nicosulfuron. (**C**) *P450-N-Z1* transgenic *Arabidopsis* challenged with penoxsulam. (**D**) *P450-N-Z1* transgenic *Arabidopsis* challenged with mesotrione. (**E**) *P450-N-Z1* transgenic *Arabidopsis* challenged with dicamba. (**F**) *P450-N-Z1* transgenic *Arabidopsis* challenged with bentazon. (**G**) *P450-N-Z1* transgenic *Arabidopsis* challenged with atrazine. In each set, the transgenic plants were in the top and the non-transgenic control plants were in the bottom.

To test if the two cloned cytochrome P450 genes actually confer herbicide tolerance, T-DNA vectors containing the expression cassette of *P450-N-Z1* or *P450-N-Z2* under the control of CaMV35S promoter and a cassette of glyphosate-tolerant enolpyruvate 5-enolpyruvylshikimate-3-phosphate synthase (EPSPS) gene from *Pseudomonas putida* as the selection marker, were constructed (Figure 1A), and then transformed into *Arabidopsis thaliana* by the *Agrobacterium*-mediated transformation method. About 20 transgenic events from each construct were generated.

**Figure 2:**
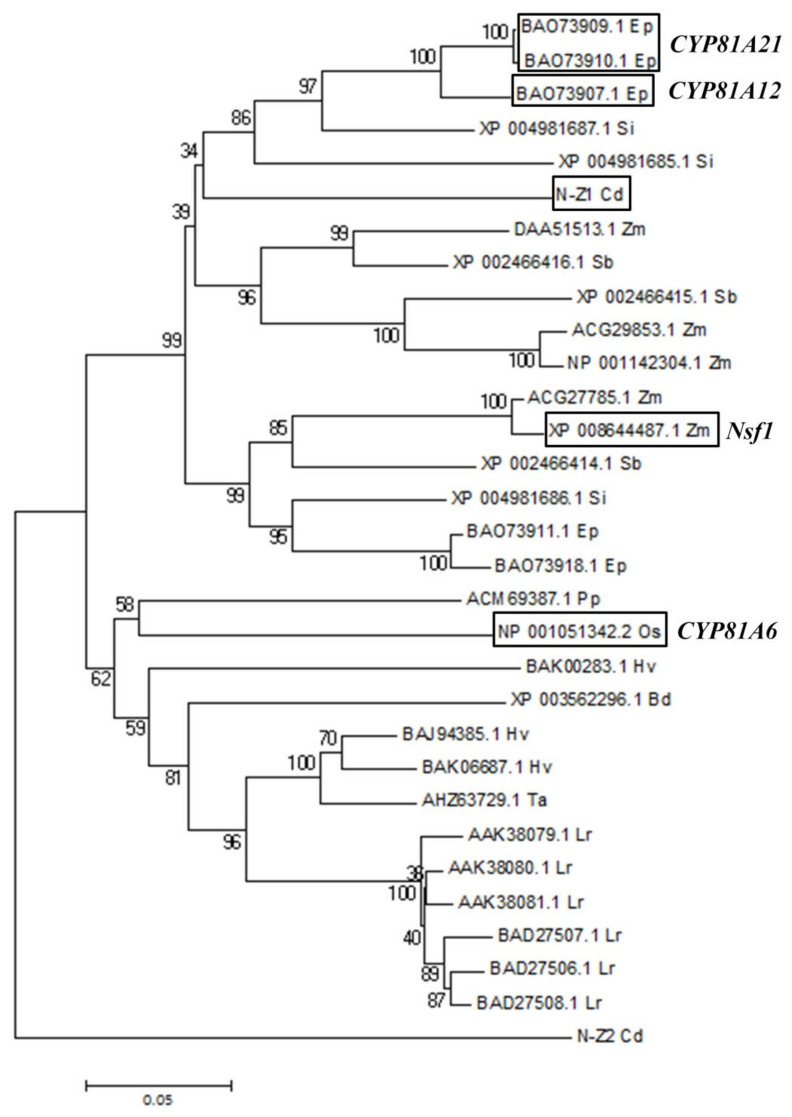
Phylogenetic analysis of P450-N-Z1 and its highly similar P450 proteins from different species. The dendrogram was constructed by the neighbor-joining method of MEG5. Ep, Si, X, Zm, Sb, Pp, Os, Hv, Bd, Ta, and Lr suffixes denote P450s from *Echinochloa phyllopogon*, *Setaria italic*, *Cynodon dactylon*, *Zea mays*, *Sorghum bicolor*, *Phyllostachys praecox*, *Oryza sativa*, *Hordeum vulgare*, *Brachypodium distachyon*, *Tritium aestivum*, and *Lolium rigidum*, respectively. The genes with known herbicide tolerance activity are highlighted by boxes.

**Figure 3:**
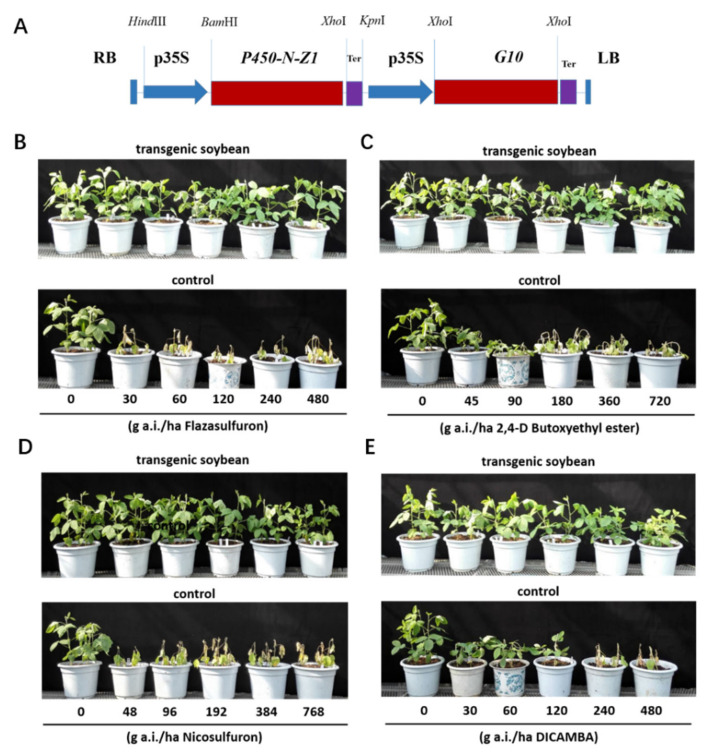
Herbicide tolerance assays of soybean expressing *P450-N-Z1*. (**A**) The diagram of the T-DNA for transformation. (**B**) Transgenic soybean sprayed with flazasulfuron. (**C**) Transgenic soybean sprayed with 2,4-D. (**D**) Transgenic soybean sprayed with nicosulfuron. (**E**) Transgenic soybean sprayed with dicamba. For each herbicide, five different concentrations were applied to both transgenic plants (**upper** panel) and non-transgenic plants (**lower** panel).

**Figure 4:**
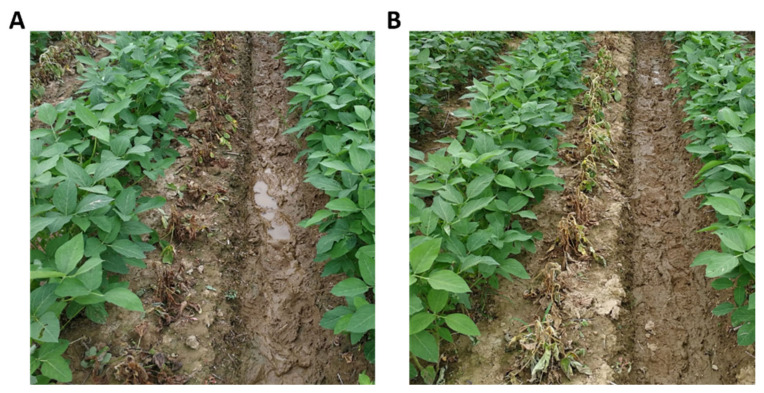
Field assays of herbicide tolerance for the transgenic soybean expressing P450-N-Z1. (**A**) Spray of flazasulfuron at 300 g a.i./ha; (**B**) spray of 2,4-D at 1440 g a.i./ha. Transgenic and non-transgenic soybean were planted and sprayed in twin rows.

The transgenic *Arabidopsis* plants were assayed for their tolerance to some commonly used commercial herbicides of different classes. We found that most of the transgenic events expressing *P450-N-Z1* showed significant tolerance to the following herbicides: (1) acetolactate synthase (ALS) inhibitor herbicides nicosulfuron and penoxsulam (Figure 1B,C); (2) p-hydroxyphenylpyruvate dioxygenase (HPPD)-inhibiting herbicide mesotrione (Figure 1D); (3) synthetic auxin herbicide dicamba (Figure 1E); (4) photosynthesis inhibitors bentazon and atrazine (Figure 1F,G). Extensive screening of more herbicides with the transgenic plants indicated that they also conferred various levels of tolerance to flazasulfuron, 2,4-D, penoxsulam, acetochlor, bensulfuron methyl, and napropamide (Figure S1).

However, none of the 20 *Arabidopsis* events transformed with *P450-N-Z2* showed any significantly increased tolerance over the non-transgenic control plants to any one of the above mentioned herbicides. Therefore, we concluded that only *P450-N-Z1* is capable of conferring tolerance to herbicides, while *P450-N-Z2* is not. The tolerance spectrum of herbicides that *P450-N-Z1* confers is almost the same as that of this gene’s host, the Bermuda grass. Thus, *P450-N-Z1* is likely the gene that is responsible for the wide spectrum tolerance of Bermuda grass to herbicides. However, we cannot rule out yet that other Bermuda grass genes may also contribute to its robust herbicide tolerance directly or indirectly.

### 2.2. Sequence Analysis of the P450 Gene Conferring Herbicide Tolerance

Sequence alignment suggested that *P450-N-Z1* and *P450-N-Z2* encode proteins which are highly similar to each other (Figure S2). BLAST search suggested that P450-N-Z1 and P450-N-Z2 are highly similar to the known P450s from plant species of Gramineae. The amino acid sequence of P450-N-Z1 shares high similarity to numerous plant P450s from various plants of Poaceae, some of which are known to be involved in herbicide detoxification, though with quite different herbicide specificity. A phylogenetic dendrogram generated among these P450s from *Echinochloa phyllopogon*, *Setaria italic*, *Cynodon dactylon*, *Zea mays*, *Sorghum bicolor*, *Phyllostachys praecox*, *Oryza sativa*, *Hordeum vulgare*, *Brachypodium distachyon*, *Tritium aestivum,* and *Lolium rigidum* showed that the known herbicide-tolerant P450s were not clustered together, but were spread in different branches (Figure 2), indicating that the P450s that confer herbicide tolerance may not be derived from a distinct P450 lineage. Thus, one may not be able to predict the herbicide tolerance capability of a plant cytochrome P450 just based on its sequence similarity. In fact, when P450-N-Z1 is compared to the P450 genes available in the GenBank, the homologous sequences that share the highest identity were not known to have the activity to confer herbicide tolerance (Table S1). 

### 2.3. Creation of Transgenic Herbicide-Tolerant Soybean

To develop transgenic soybean plants tolerant to multiple herbicides, we constructed a binary vector pRF028 carrying a T-DNA containing the *P450-N-Z1* expression cassette under control of CaMV35S promoter and a glyphosate-tolerant EPSPS gene *G10* cassette from *Deinococcus radiodurans* (GenBank ATK09487.1) as selection marker (Figure 3A), and then transformed the local elite soybean line Tianlong-1 by the *Agrobacterium*-mediated transformation method.

About 200 independent soybean events in total were generated with vector pRF028. The presence of *P450-N-Z1* was verified by gene-specific PCR analysis of T0 events. The soybean events were screened by a spray of flazasulfuron at the V4–V5 stage at T1 generation. Over half of the transgenic soybean events showed different levels of tolerance to flazasulfuron. The expression of P450-N-Z1 in the leaves of flazasulfuron-tolerant transgenic soybean was demonstrated by Western blot analysis (Figure S3) and quantified by ELISA (Table S2). One event, G3X-3, carrying a single copy of T-DNA insertion, was characterized and selected for further tests of tolerance to various herbicides in the greenhouse at T2 (Figure 3B–E).

The transgenic soybean is highly tolerant to flazasulfuron. It showed no significant damage at a dose of 480 g active ingredient (a.i.)/ha, while the non-transgenic soybean plants were killed at less than 30 g a.i./ha. Flazasulfuron is a powerful herbicide and is active against a wide range of annual and perennial broadleaf weeds, grasses, and sedges. It was registered for use on warm season turf grass, grapevine, sugarcane, and non-crop areas in various countries, and may be used both pre-emergence and post-emergence. The transgenic soybean is tolerant to at least 6.4 times the recommended rate, which is 37.5~75 g a.i./ha for weed control of turf grasses and sugarcane.

The transgenic soybean also demonstrated high tolerance to nicosulfuron, which is a broad spectrum herbicide that controls a wide range of annual and perennial broadleaf weeds, grasses, and sedges. It is currently primarily used for weed control in corn fields. The transgenic soybean is tolerant to nicosulfuron at about 384 g a.i./ha without significant damage, which is much higher than the recommended rate for corn fields, 40~60 g a.i./ha.

The transgenic soybean also showed good tolerance to 2,4-D. No significant plant injury was observed up to 720 g a.i./ha, which is the recommended rate for corn field application. Higher rates resulted in visible injuries, but the plants mostly recovered in 3 to 4 days.

The transgenic soybean showed significantly improved tolerance to dicamba. While the growth of non-transgenic soybean was inhibited by dicamba at the rate of 30 g a.i./ha, the transgenic soybean was not visibly injured by dicamba at rates up to 240 g a.i./ha. Rates at 480 g a.i./ha resulted in visible injuries to the transgenic soybean. The recommended rate for dicamba is 560 g a.i./ha.

### 2.4. Field Evaluation of Transgenic Soybean

To evaluate the efficacy of the transgenic soybean in field conditions, we carried out a field trial in 2017 in the Experiment Farm of Zhejiang University at Changxing, Zhejiang province, China. The transgenic line G3X-3 and the non-transgenic control were both sprayed with flazasulfuron at rates of 75 g a.i./ha, 150 g a.i./ha, and 300 g a.i./ha (1×, 2×, 4× of recommended application rate) and 2,4-D at rates of 360 g a.i./ha, 720 g a.i./ha, and 1440 g a.i./ha (0.5×, 1×, 2× of recommended application rate) at V4~V5 stage. No visible injuries were observed in all the dosages of both herbicides. The field trial demonstrated that the transgenic soybean is highly tolerant to flazasulfuron and 2,4-D, two of our preferred herbicides for field application (Figure 4).

### 2.5. Transgenic Soybean’s Capability of Metabolizing Herbicides In Vivo

**Figure 5:**
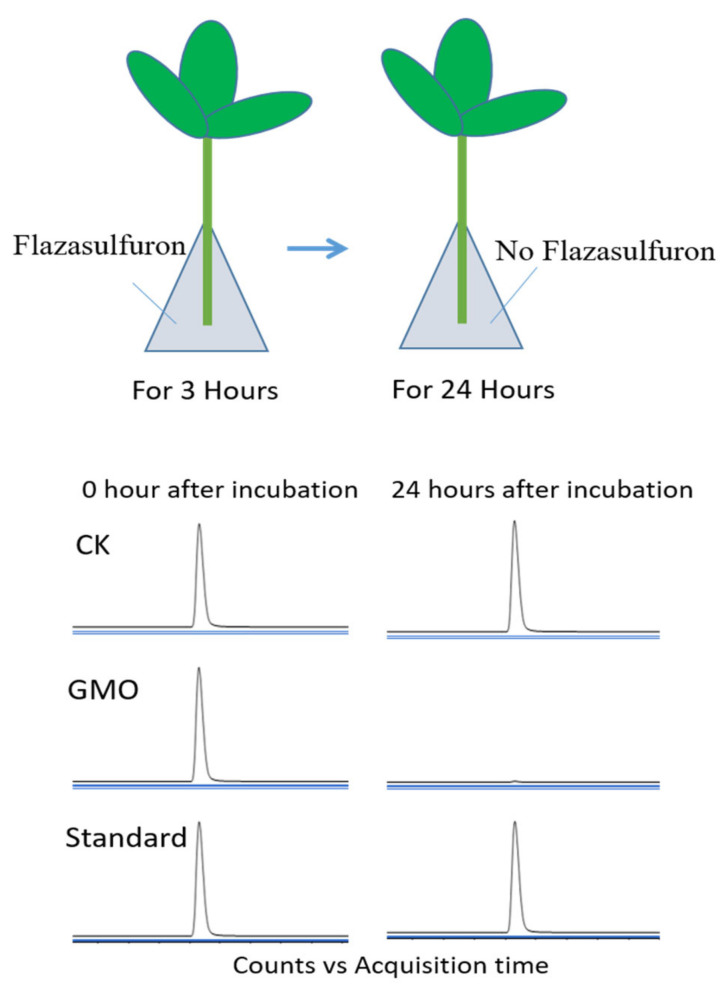
Flazasulfuron was rapidly degraded by transgenic soybean expressing P450-N-Z1. Upper panel: diagram showing that the soybean leaves were cut to be cultured with solution containing flazasulfuron for 3 h, and then with solution containing no flazasulfuron for 24 h. Lower panel: the flazasulfuron in the leaves was detected by HPLC chromatograms at 0 h and 24 h after herbicide incubation. CK, non-transgenic soybean as control; GMO, transgenic soybean; Standard, chemical standard of flazasulfuron.

We postulated that P450-N-Z1 confers herbicide tolerance by degrading the herbicides. To confirm this experimentally, we compared the rate of degradation of flazasulfuron and dicamba in the transgenic soybean G3X-3 and the non-transgenic soybean. We cultured the cut leaves in a solution containing herbicides for 3 h and then moved to a solution containing no herbicides to continue to culture for 24 h. The herbicides within the leaves after culture were measured by high-performance liquid chromatography tandem mass spectrum (HPLC-MS/MS). We found that the transgenic leaves had metabolized 99.5% of flazasulfuron after 24 h culture, while the non-transgenic recipient soybean control only degraded 24.9% (Figure 5). For dicamba, it was 92.5% degradation in transgenic soybean and 21% in non-transgenic control plant leaves (Figure S3). This experiment demonstrated that the *P450-N-Z1* transgenic soybean is capable of degrading the herbicides rapidly. Moreover, the results suggested that it removed flazasulfuron faster than dicamba, which is in agreement with the observation that the transgenic soybean is more robust in tolerance to flazasulfuron than dicamba.

## 3. Discussion

### 3.1. Potential Role of P450-N-Z1 in Bermuda Grass

Bermuda grass is known to be tolerant to quite a number of selective herbicides, including nicosulfuron and flazasulfuron. However, little is known about the underlying molecular mechanism. It was only recently that the first genome of the *Cynodon* genus, *C. transvaalensis*, was published [33]. We hypothesized that the wide spectrum of herbicide tolerance in Bermuda grass may be attributed to *CYP450s* in the *CYP81A* family, since *CYP81As* were shown to confer herbicide tolerance to various herbicides. Of the two *CYP81A* homologs cloned from Bermuda grass, heterologous expression in *Arabidopsis* showed that *P450-N-Z1* was able to confer herbicide tolerance to herbicides of four different classes: ALS inhibitor herbicides nicosulfuron and penoxsulam, HPPD-inhibiting herbicide mesotrione, synthetic auxin herbicides 2,4-D and dicamba, and photosynthesis inhibitor bentazon. It is very likely that *P450-N-Z1* is involved in the tolerance of Bermuda grass to the wide spectrum of herbicides. However, it is not certain whether *P450-N-Z1* is the sole gene responsible for the wide spectrum of herbicide tolerance in Bermuda grass. Works in tobacco, rice, *E. phyllopogon*, and *L. rigidum* have revealed that one herbicide may be metabolized by different P450s in a single species, and different herbicides may be metabolized by different P450s within a single species. In tobacco, chlortoluron is metabolized by CYP71A11 via *m*-methyl hydroxylation and CYP81B2 via N-demethylation and *m*-methyl hydroxylation [15]. In rice, bensulfuron methyl is detoxified by both CYP81A6 and CYP72A31, whereas detoxification of bentazon is mainly mediated by CYP81A6 and bispyribac sodium by CYP72A31 [21,23]. In *E. phyllopogon*, both CYP81A12 and CYP81A21 are involved in bensulfuron methyl and penoxsulam detoxification. In *L. rigidum*, chlortoluron is metabolized by CYP71R4, while CYP81A10v7 metabolizes five different classes of herbicides including chlortoluron [29,34]. To our knowledge, there is no *P450-N-Z1* mutant Bermuda grass currently available. It would be helpful to generate *P450-N-Z1* knockout Bermuda grass by CRISPR technology and test the KO to identify if other P450s are involved in the detoxification of the herbicides metabolized by P450-N-Z1.

Sequence analysis showed that P450-N-Z1 shares high similarity with P450s from the CYP81A family. After consulting with the P450 nomenclature committee, P450-N-Z1 and P450-N-Z2 were assigned as CYP81A69 and CYP81A70, respectively [35]. *CYP81As* are specific to the Poaceae lineage. The number of *CYP81As* varies between species, ranging from as few as 2 in *B. distachyon* and as many as 11 in *Z. mays* [12]. We were able to clone two *CYP81As* from Bermuda grass in the present research. However, due to the lack of Bermuda grass genome data, it is not known whether there are other *CYP81As* in Bermuda grass. P450-N-Z1 shares 66.4% sequence identity with P450-N-Z2, 71.9% identity with rice CYP81A6, and 75.5% identity with corn CYP81A9. Compared with P450-N-Z2, P450-N-Z1 shares more similarity with the homologous proteins known to confer herbicide tolerance in rice and corn. Given the high similarity of P450-N-Z1 to CYP81A6 and CYP81A9, it is not very surprising that *P450-N-Z1* confers tolerance to a broad spectrum in *Arabidopsis* while *P450-N-Z2* does not.

The rapid degradation of flazasulfuron and dicamba in transgenic soybean provided direct evidence supporting the hypothesis that herbicide tolerance conferred by *P450-N-Z1* was achieved by metabolism of the herbicides. In our results, 24 h post-treatment, flazasulfuron residue in transgenic soybean leaf was 0.5% compared to 75.1% in non-transgenic soybean; dicamba residue in transgenic soybean leaf was 7.5% compared to 79% in non-transgenic soybean. In a similar study of transgenic rice overexpressing *L. rigidum* CYP81A10v7, diclofop acid residue was 23.4% to 29.0% in transgenic lines compared to >50% in GFP transgenic rice controls 48 h post-treatment [29]. The low residue of herbicides detected within a short time post-spray in transgenic soybean suggests that P450-N-Z1 is a highly potent P450.

Although *P450-N-Z1* was demonstrated to confer herbicide tolerance to transgenic *Arabidopsis* and soybean, the reactions catalyzed by P450-N-Z1 in Bermuda grass remain unknown. Bermuda grass, in addition to being a weed, also serves as cattle feed and turf grass. There has been no report on the evolution of herbicide resistance in Bermuda grass so far. The broad spectrum of herbicide tolerance in Bermuda grass is likely conferred by *P450-N-Z1* fortuitously. Thus, it is of interest to understand the role of P450-N-Z1 in Bermuda grass’s secondary metabolism and how the pathways are regulated.

### 3.2. Utilization of P450-N-Z1 for Biotechnology

First, *P450-N-Z1* can be a valuable tool for plant transformation. A previous study has demonstrated that rice *CYP81A6* confers tolerance to bentazon and bensulfuron-methyl in *Arabidopsis* and tobacco, serving as a selection marker for transgenic plants [22]. We demonstrated that expression of *P450-N-Z1* confers herbicide tolerance in both model plant *A. thaliana* and broadleaf crop soybean. In theory, any *CYP450* demonstrated to confer high herbicide tolerance in *Arabidopsis* can be utilized as a marker for positive transgenic *Arabidopsis* selection. Furthermore, in transformation systems where plant tissue culture is required, such as soybean and corn, it is desirable to develop novel selection markers and use selection reagents at low cost. Initial trials in soybean and corn transformation have been successful in our lab using *P450-N-Z1* as the selection marker and flazasulfuron as the selection reagent, although the concentration of flazasulfuron in the selection medium needs further optimization.

Second, *P450-N-Z1* can be utilized to develop herbicide-tolerant crops. Quite a few P450 genes have previously been identified to confer tolerance to various herbicides (Table S3). These P450 genes are active against different herbicides, and with different efficacy. However, a preferred plant P450 gene to be utilized for engineering transgenic crops needs to have robust activity against herbicides with a wide spectrum of herbicidal activity. Our research showed that *P450-N-Z1* can be a preferred plant P450 gene for engineering transgenic herbicide-tolerant crops. Transgenic soybean expressing *P450-N-Z1* is highly tolerant to a wide spectrum of herbicides, especially, flazasulfuron and nicosulfuron, both of which are highly effective herbicides for control of broadleaf weeds and grasses.

Although the amino acid sequence of P450-N-Z1 is highly similar to the herbicide detoxification P450s Nsf1 (XP_008644487) from corn and CYP81A6 (NP_001051342) from rice, it differs from them significantly in the tolerance spectrum of herbicides. Nsf1 was found to confer tolerance to nicosulfuron and bentazon [24,25], but not to flazasulfuron, while the rice CYP81A6 has good tolerance to bentazon and some sulfonylurea herbicides [21,22], but not to flazasulfuron and nicosulfuron. 

In this study, we created a transgenic soybean expressing P450-N-Z1 and G10-EPSPS. Our study demonstrated that this transgenic soybean is highly tolerant to flazasulfuron, nicosulfuron, and 2,4-D, in addition to glyphosate, which results from expression of the selection marker gene *g10-epsps*. With this transgenic soybean, we will have more options for selecting herbicides to control weeds. In addition to the single use of the above mentioned herbicides, combinations of two or three herbicides may be used to control weeds. One such option is to use combinations of glyphosate and flazasulfuron. A commercial herbicide mixture of glyphosate and flazasulfuron named Chakara Duo (Belchim Crop Protection) is claimed to be more effective than either individual herbicide in the control of hard-to-kill weeds. Furthermore, flazasulfuron or its mixture may be used either as post- or pre-emergency herbicide, and thus offers a longer time frame for application. 

The transgenic soybean created by this study is also dicamba-drifting-proof. In the past couple of years, spray of dicamba in dicamba-tolerant soybean fields in the USA caused significant damage to neighbor soybean plants with no dicamba trait due to the issue of dicamba drifting. The transgenic soybean we created does not show robust tolerance to dicamba to serve as a transgenic trait, but it is quite sufficient to tolerate any level of drifting dicamba. Thus, this transgenic soybean is compatible with the dicamba-tolerant soybean.

## 4. Materials and Methods

### 4.1. Cloning of P450 Genes from C. dactylon

Two PCR primers 450F (5′ACGGCCCGTACTGGCGCAACCTCCGCCG) and 450R (5′GTTCCTCA CGCCGAACACGTCGAACCACCG) were designed according to the consensus sequences of P450 genes from closely related species. Total mRNA was extracted from *C. dactylon* with TRIzol reagent (Invitrogen, Carlsbad, CA, USA), and cDNA was synthesized with oligo-dT primer 3′DTANCHOR using One Step RT-PCR kit (Qiagen, Venlo, The Netherlands). PCR was carried out using the cDNA as template with primers 450F and 450R. The procedure of PCR is: 95 °C for 1 min, 58 °C for 1 min, 72 °C for 1 min, repeated for 30 cycles, then 72 °C for 5 min. The PCR products were cloned into pMD18-T (Shanghai Sangon, Shanghai, China) and sequenced. Using the partial sequences of the cDNA, the full-length cDNA sequences were then obtained using the SMARTer RACE 5′/3′ Kit (TaKaRa Bio, Kusatsu, Japan). 

Based on the sequencing results of cDNA, the two full-length cDNAs were amplified by PCR using primer P450-N (5′GGATCCAACAATGGATAAGGCCTACGTGGC) and P450-N-Z1-R (5′CTCGAGTCAGAGCTCCTGCAAAACCTCAC), and Premier P450-N and P450-N-Z2-R (5′CTCGAGTCATTACGCGAGCAGCCCCTTGAG), respectively. The two cDNA share the same 5′ end primers. PrimeSTAR HS DNA polymerase (TaKaRa Bio, Japan) was used for PCR, and the PCR procedure was 32 cycles of 98 °C for 10 s, 66 °C for 2 min, then 72 °C for 10 min. The PCR products were cloned into pMD18-T and sequenced.

### 4.2. Construction of the T-DNA Vectors for Arabidopsis Transformation

The binary T-DNA transformation plasmid was built based on pCAMBIA1300. First, an intermediate vector pCAMBIA1300-G6 was constructed by substituting the hygromycin-resistant gene for the glyphosate-resistant 5-enolpyruvylshikimate-3-phosphate synthase (EPSPS) gene *G6* (GI: 8469109) between *Xho*I sites. Then we constructed the expression cassettes of *P450-N-Z1* or *P450-N-Z2* composed of the CaMV 35S promoter, *P450-N-Z1* or *P450-N-Z2* gene and the CaMV 35S terminator with a *Hind*III site at the 5′ end and a *Kpn*I site at the 3′ end, respectively. The expression cassettes were inserted between the *Hind*III site and *Kpn*I site of the intermediate vector pCAMBIA1300-G6 to obtain the transformed vectors pCAMBIA1300-G6-P450-N-Z1 and pCAMBIA1300-G6-P450-N-Z2, respectively (Figure 1A).

### 4.3. Arabidopsis Transformation

The two binary vectors pCAMBIA1300-G6-P450-N-Z1 and pCAMBIA1300-G6-P450-N-Z2 with glyphosate as the selection agent were introduced into electrocompetent *A. tumefaciens* strain LBA4404 using an Electroporator 2510 (Eppendorf) following the manufacturer’s instructions. The presence of the T-DNA in selected *Agrobacterium* colonies was verified with PCR analysis using vector-specific primers. *Arabidopsis* was transformed with the floral dip method [36] using the transformed *A. tumefaciens* strain LBA4404.

### 4.4. Construction of the T-DNA Vector for Soybean Transformation

The binary T-DNA vector pRF028 for soybean transformation was constructed based on pCAMBIA1300. Firstly, an intermediate vector pCAMBIA1300-G10 was constructed by substituting the hygromycin-resistant gene for the glyphosate-resistant 5-enolpyruvylshikimate-3-phosphate synthase (EPSPS) gene *G10* (ATK09487.1) between *Xho*I sites. Then we constructed the expression cassettes of *P450-N-Z1* composed of the CaMV 35S promoter, *P450-N-Z1* gene, and the CaMV 35S terminator with a *Hind*III site at the 5′ end and a *Kpn*I site at the 3′ end, respectively. These expression cassettes were cloned between the *Hind*III site and *Kpn*I sites of the intermediate vector pCAMBIA1300-G10 to obtain the transformation vector pCAMBIA1300-G10-P450-N-Z1 (Figure 3A).

### 4.5. Soybean Transformation

The binary vector pRF028 was introduced into *Agrobacterium tumefaciens* strain EHA105 by electroporation. Soybean transformation was carried out by *Agrobacterium*-mediated transformation of soybean half-seed cotyledonary explants [37]. Briefly, disinfected Tianlong-1 soybean seeds were soaked in sterile distilled water overnight. Cotyledonary explants were isolated and inoculated with *Agrobacterium* for 30 min. After inoculation, explants were cocultivated for 5 days. Shoot initiation medium was supplemented with 200 mg/L timentin for removal of *Agrobacterium* and 30 mg/L glyphosate for selection of transformed shoots. Shoot elongation medium was supplemented with 10 mg/L glyphosate. Elongated shoots were dipped in 1 mg/L IBA and transferred to rooting medium. Rooted plantlets were transplanted to soil mix. T0 plants were screened by PCR analysis for the presence of *P450-N-Z1* gene.

### 4.6. Characterization of Herbicide Tolerance in Transformed Arabidopsis

The transformed *Arabidopsis* seeds (obtained after the germination of T0 generation seeds) were planted, and the T1 plants, at two to four leaf-growth stages (10 d after planting (DAP)), were sprayed with glyphosate at the rate of 900 g a.i./ha to remove the null segregants. Survivors (plants actively growing) were identified 5–7 d after spraying and were sprayed with penoxsulam (at the rate of 40 g a.i./ha; the recommended application rate for rice fields is 30–60 g a.i./ha), acetochlor (at the rate of 360 g a.i./ha; the recommended application rate for corn fields is 900–1125 g a.i./ha), bensulfuron methyl (at the rate of 40 g a.i./ha; the recommended application rate for rice fields is 27–45 g a.i./ha), flazasulfuron (at the rate of 40 g a.i./ha; the recommended application rate for turf grass is 37.5–75 g a.i./ha), 2,4-D (at the rate of 280 g a.i./ha; the recommended application rate for corn fields is 720 g a.i./ha), napropamide (at the rate of 600 g a.i./ha; the recommended application rate for cotton fields is 1100–1800 g a.i./ha), nicosulfuron (at the rate of 24 g a.i./ha; the recommended application rate for nicosulfuron in corn fields is 40–60 g a.i./ha), penoxsulam (at the rate of 40 g a.i./ha; the recommended application rate for rice fields is 30–60 g a.i./ha), mesotrione (at the rate of 60 g a.i./ha; the recommended application rate for corn fields is 105–150 g a.i./ha), dicamba (50 g a.i./ha; the recommended application rate for corn fields is 560 g a.i./ha), bentazon (600 g a.i./ha; the recommended application rate for soybean fields is 1.1–1.4 kg a.i./ha), and atrazine (600 g a.i./ha; the recommended application rate for corn fields is 1.1–1.4 g a.i./ha), respectively, to identify the tolerance ability. Those plants with the highest tolerance were propagated and subsequently challenged with the following herbicides with various rates at four to six leaf-growth stages including: nicosulfuron (at rates between 32 and 128 g a.i./ha; the recommended application rate for corn fields is 40–60 g a.i./ha), penoxsulam (at rates between 10 and 40 g a.i./ha; the recommended application rate for rice fields is 30–60 g a.i./ha), mesotrione (at rates between 80 and 320 g a.i./ha; the recommended application rate for corn fields is 105–150 g a.i./ha), dicamba (at rates between 30 and 120 g a.i./ha; the recommended application rate for corn fields is 560 g a.i./ha), bentazon (at rates between 1 and 4 kg a.i./ha; the recommended application rate for soybean fields is 1.1–1.4 kg a.i./ha), and atrazine (0.6–2.4 kg a.i./ha; the recommended application rate for corn fields is 1.1–1.4 g a.i./ha). 

### 4.7. Characterization of Herbicide Tolerance in Transformed Soybean

Seeds of T0 soybean plants were planted in 5-gallon pots. A spray of flazasulfuron at a rate of 150 g a.i./ha at the stage of V3–V4 was applied in a handheld sprayer. Tolerance to flazasulfuron was visually rated. Event G3X-3 bearing a single copy of T-DNA insertion and good tolerance to flazasulfuron was characterized and advanced to T2 for tests with different herbicides.

Seeds of transgenic soybean G3X-3 T1 plants and non-transgenic control were planted in 5-gallon pots (4 plants per pot) and sprayed with various herbicides at various rates at V4 stage, including flazasulfuron (at rates between 30 and 480 g a.i./ha; the recommended application rate for turf grass is 37.5–75 g a.i./ha), nicosulfuron (at rates between 48 and 768 g a.i./ha; the recommended application rate for corn fields is 40–60 g a.i./ha), 2,4-D (at rates between 45 and 720 g a.i./ha; the recommended application rate for corn fields is 720 g a.i./ha), and dicamba (at rates between 30 and 480 g a.i./ha; the recommended application rate for corn fields is 560 g a.i./ha).

### 4.8. Sequence Analysis of the P450 Gene Conferring Herbicide Tolerance

Blastp search of P450-N-Z1 in the NCBI non-redundant protein database with parameters set to default was conducted. The search found P450s with high similarity to P450-N-Z1 from the following species: *Echinochloa phyllopogon*, *Setaria italic*, *Cynodon dactylon*, *Zea mays*, *Sorghum bicolor*, *Phyllostachys praecox*, *Oryza sativa*, *Hordeum vulgare*, *Brachypodium distachyon*, *Tritium aestivum*, and *Lolium rigidum*. P450 polypeptide sequences from these species were download with the following accessions: *Echinochloa phyllopogon* BAO73909.1, BAO73910.1, BAO73907.1, BAO73911.1, BAO73918.1; *Setaria italic* XP 004981685.1, XP 004981686.1, XP 004981687.1; *Zea mays* DAA51513.1, ACG29863.1, NP001142304.1, ACG27785.1, XP 008644487.1; *Sorghum bicolor* XP 002466414.1, XP 002466415.1, XP 002466416.1; *Phyllostachys praecox* ACM69387.1; *Oryza sativa* NP 001051342.2; *Hordeum vulgare* BAK00283.1, BAJ94385.1, BAK06687.1; *Brachypodium distachyon* XP 003562296.1; *Tritium aestivum* AHZ63729.1; *Lolium rigidum* AAK38079.1, AAK38080.1, AAK38081.1, BAD27506.1, BAD27507.1, BAD27508.1. The dendrogram was constructed by the neighbor-joining method of MEGA5.

### 4.9. Western Blot Analyses

The 6th fully developed trifoliate leaf of T1 soybean events was collected at V7–V8 stage. First, 30 mg leaf tissue was weighed and ground to a fine powder in the presence of liquid nitrogen. Total protein was extracted with 300 μL protein extraction buffer (50 mM Tris-HCl pH 7.4, 150 mM NaCl, 1% Triton X-100, 1% sodium deoxycholate, 0.1% SDS). After lysis and centrifugation, the supernatant containing the soluble proteins was mixed with 5X protein-loading buffer and boiled for 10 min. The samples were subjected to centrifugation at 12,000× *g* for 5 min and 10 μL of sample was loaded onto 4–20% surePAGE^TM^ precast gel (Genscript), and then transferred onto polyvinylidene fluoride (PVDF) membranes using eBlot^TM^ L1 transfer system (Genscript). After blocking in 5% skim milk in PBST for 1 h at room temperature, mouse monoclonal antibody against P450-N-Z1 was used as the primary antibody. Goat Anti-Mouse IgG (H&L) (HRP) was used as the secondary antibodies. The bands were visualized by enhanced chemiluminescence (ECL) substrates.

### 4.10. ELISA Analysis of Transgenic Soybean

The 6th fully developed trifoliate leaf of T1 soybean events was collected at V7–V8 stage. First, 30 mg leaf tissue was weighed and ground to a fine powder in the presence of liquid nitrogen. Total protein was extracted with 1 mL protein extraction buffer (50 mM Tris-HCl pH 7.4, 150 mM NaCl, 1% Triton X-100, 1% sodium deoxycholate, 0.1% SDS). After lysis and centrifugation, the supernatant containing the soluble proteins was diluted with phosphate-buffered saline (PBS, pH 7.4) by a factor of 100-fold. The diluted solutions were used for ELISA analysis. In total, 100 μL of P450-N-Z1 specific monoclonal antibody (5 μg/mL) was immobilized on a microtiter well to capture P450-N-Z1 protein in plant extracts. To prepare a standard curve, different concentrations of purified P450-N-Z1 proteins (8, 4, 2, 1, 0.5, and 0.25 ng/mL) were added into the microtiter wells on the same assay plate. The plate was incubated for 1 h with shaking at 200 rpm at ambient temperature and washed four times with PBS with 0.05% Tween 20 (PBST). Biotinylated anti–P450-N-Z1 monoclonal antibody (1 μg/mL) solution in PBST was added and incubated for 1 h to detect the bound P450-N-Z1 protein. After another wash step with PBST, neutravidin–horse reddish peroxidase (HRP) conjugate was added to each well and incubated for another 1 h. After another wash step, 3,3′,5,5′-Tetramethylbenzidine (TMB) substrate (100 μL) was added, and after 15 min of incubation, 100 μL stop solution (1 N HCl) was added to stop the reaction. The absorbance at 450 nm was measured using a microtiter plate reader. The concentration of proteins in plant extracts was extrapolated from the standard curve developed on the same plate.

### 4.11. Field Trials of the Transgenic Soybean

The field trial was conducted with permits from regulators in China at Zhejiang University Farm at Changxing, Zhejiang Province in the regular soybean-growing season in 2017. Homozygous T4 generation seeds of transgenic soybean G3X-3 and non-transgenic soybean seeds were planted side by side in 8 m × 1 m twin rows. The plants were sprayed with either flazasulfuron or 2,4-D at V4–V5 stage. Flazasulfuron was applied at rates of 75 g a.i./ha, 150 g a.i./ha, and 300 g a.i./ha (1×, 2×, 4× of recommended application rate). 2,4-D was sprayed at rates of 360 g a.i./ha, 720 g a.i./ha, 1440 g ae/L (0.5×, 1×, 2× of recommended application rate).

### 4.12. In Vivo Herbicide Degradation Study

Analytical grade standard flazasulfuron (99.9%) and dicamba (99.9%) were purchased from Fluka (Sigma-Aldrich, USA). HPLC grade acetonitrile was purchased from Merck (Merck Chemicals, Germany); other analytical reagent chemicals were supplied by Tjshield (Shield Company, Tianjin, China). In total, 25% water-dispersible granule of flazasulfuron (ISK Biotech, Tokyo, Japan) and 48% aqueous solution of dicamba (Shenghua Biok Chemical, Huzhou, China) were used for the herbicide degradation assays.

The experiments were conducted in a greenhouse while the transgenic soybean line G3X-3 and non-transgenic control soybean were in the third to the fourth compound leaf of seedling stage. The third compound leaves of soybean removed from G3X-3 and non-transgenic control were dipped into the 100 mg/L flazasulfuron or 400 mg/L dicamba solutions, respectively, for 3 h to absorb herbicides. The petioles were washed to ensure that unabsorbed herbicide residues were removed, and then leaves were transferred to nutrient solution for mimicking the in vivo metabolism. The G3X-3 and non-transgenic control soybean leaves were harvested at 0 and 24 h after treatment, and all the leaf samples were milled by liquid nitrogen for further analysis.

Flazasulfuron powdered sample was extracted with acetonitrile-phosphate buffer (2:8 V/V, pH 7.8) under ultrasonic bath for 15 min. The homogenized sample was filtered by Whatman filter paper. The extract was evaporated to dryness by rotary evaporation and dissolved in mobile phase solution [38]. Dicamba sample was extracted with V(acetonitrile):V(formic acid):V(H_2_O) = 49.5:0.2:0.3 by ultrasonic extraction for 15 min then salted out by sodium chloride. After filtration, the extract was concentrated by rotary evaporator. The final residue was dissolved in mobile phase solution [39].

### 4.13. LC-MS/MS Analysis

All the samples were analyzed by HPLC- MS/ MS on an Agilent 6460 Triple Quad LC/MS (Agilent, Palo Alto, USA). The analytical column was an Agilent Zorbax Eclipse XDB-C18 (150 mm × 2.1 mm, 3.5 μm). The MS system was electrospray ionization (ESI) source. The mobile phases were 0.1% (*v*/*v*) formic acid (A) and 100% acetonitrile (B). The column oven was maintained at a temperature of 35 ℃. The flow rate was 0.3 mL min^−1^ and the injection volume was 5 μL.

The flazasulfuron samples were separated with the following gradient program: 0–5 min, followed by a linear gradient from 10% B to 60% B; 5–8 min, followed by a linear gradient from 60% B to 75% B; 8–10 min, followed by a linear gradient from 75% B to 95% B. The instrument was operated in positive ionization mode in the multiple reaction monitoring (MRM) mode at *m*/*z* 408 and 182 [38]. The dicamba samples were separated with the following gradient program: 0–8 min, followed by a linear gradient from 0% B to 35% B; 8–10 min, maintaining 95% B. The instrument was operated in negative ionization mode under the multiple reaction monitoring (MRM) mode at *m*/*z* 219 and 175 [39].

## 5. Patents

Hangzhou Ruifeng Biosciences holds a US patent entitled “Herbicide resistance gene and use thereof”. Named inventors are Z.S., C.Y.Lin, and C.Y.Liu.

## Supplementary Materials

The following supporting information can be downloaded at: https://www.mdpi.com/article/10.3390/plants11070949/s1, Figure S1: Herbicide tolerance tests of the *Arabidopsis* transformed with *P450-N-Z1* in greenhouse; Figure S2: Alignment of deduced amino acid sequences of *P450-N-Z1* and *P450-N-Z2*; Figure S3: Western blot analysis of transgenic events of soybean at T1; Figure S4: Dicamba was rapidly degraded by transgenic soybean expressing P450-N-Z1; Table S1: P450 genes sharing the highest sequence identity with *P450-N-Z1*; Table S2: Expression of P450-N-Z1 in transgenic soybean lines; Table S3: Expression of P450-N-Z1 in transgenic soybean lines.
