# Supplementary material for: Expression of a Cytochrome P450 Gene from Bermuda Grass Cynodon dactylon in Soybean Confers Tolerance to Multiple Herbicides
Source: Plants (Basel). 2022 Mar 31;11(7):949. doi: 10.3390/plants11070949 (PMC9002376; doi:10.3390/plants11070949)
Supplement: Supplementary file 1 [file plants-11-00949-s001.zip › plants-1616438-supplementary.pdf]

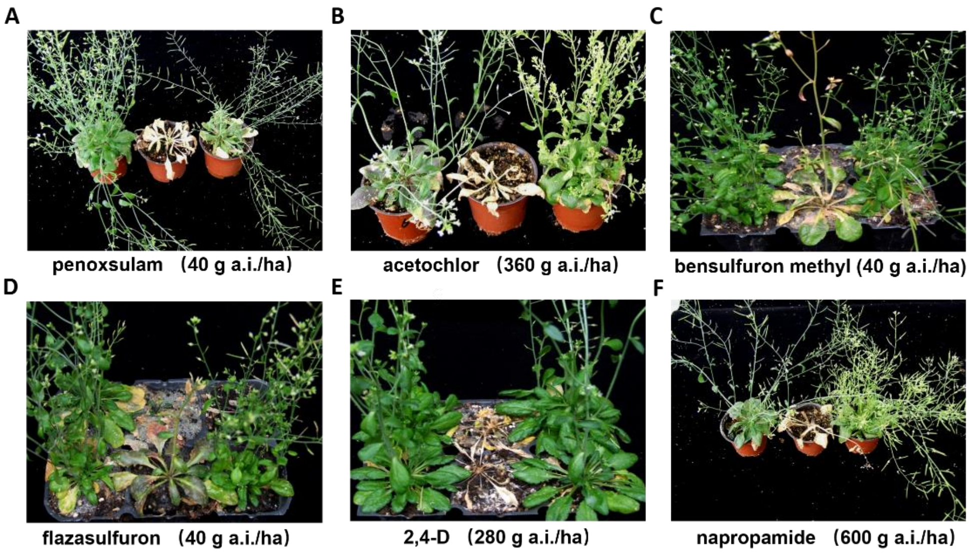

Figure S1. Herbicide tolerance tests of the *Arabidopsis* transformed with *P450-N-Z1* in greenhouse. (**A**) Penoxsulam (40 g a.i./ha); (**B**) Acetochlor (360 g a.i./ha); (**C**) Bensulfuron methyl (40 g a.i./ha); (**D**) Flazasulfuron (40 g a.i./ha); (**E**) 2, 4-D (280 g a.i./ha); (**F**) Napropamide (600 g a.i./ha). Transgenic plants are at right and left side of each panel, while the non-transgenic plants are at the middle.

```
P450-N-Z1        1 MDKAYVALLSF-ASLFLLHYLV-SRRN------GTGKGSKAKGALPPSPPSVPFLGHLHL    52
P450-N-Z2        1 MDKAYVAIVLSILFLFSIQRFLGHRRRSRSNVDNGKNKSVTHNRLPPGPRAVPVLGHLHL    60
                   *******::     ** :: ::  **.      .  : * ::. ***.* :**.******

P450-N-Z1       53 VKTPFHAALARLADCHGPVFSLRMGARPAVVVSSPEHAKECFTEHDVAFANRPRFPSQQL   112
P450-N-Z2       61 LKKPIHAALARLASQHGPLFSLRLGSRPAVVVTSAELARECFTEHDVTFATRPRFASLDL   120
                   :*.*:********. ***:****:*:******:* * *:********:**.**** * :*

P450-N-Z1      113 ASFNGAALGSASYGPYWRNLRRVATVHLLSAHRVACMTGTIAAEVRAMVRRMNRAAQVAS   172
P450-N-Z2      121 VSFGGTTLPTSRYGPYWRNLRRVATVHLLSAHRVGCMLPVVSSEVRAMARRVYRAAAAAP   180
                   .**.*::* :: **********************.**  .:::*****.**: *** .*

P450-N-Z1      173 GGAARIELKRRLFEVSLSVLMETIARTKTSRTEADDDTDMSPEAREFKQIVDELLPHLGT   232
P450-N-Z2      181 RGAARVELKRRLFELSLSALMETIARTKTSRAEADADRDMSPETQEFKEALDEFIPLIGA   240
                    ****:********:***.************:*** * *****::***: :**::* :*:

P450-N-Z1      233 ANLWDYMPVLRWFDVFGVRKKIVSAVRRRDAFLRHLVDAERTRLDDGN---DAGEKKSII   289
P450-N-Z2      241 ANVWDFLPLLRWLDVFGVRRKILAAVSRRDAFLQRLIDAERRRLGDDNSCNDGSDKKSMI   300
                   **:**::*:***:******:**::** ******::*:**** **.*.*   *..:***:*

P450-N-Z1      290 AMLLTLQKSEPDVYSDTMIMALCGNLFGAGTETTSTTTEWAMSLLLNHPEKLRKAQAEID   349
P450-N-Z2      301 AVLLNLQKTEPEVYTDATIMALCTSMFTGGAETTATTTEWAMSLLLNHPDVLKKAQAEMD   360
                   *:**.***:**:**:*: ***** .:* .*:***:**************: *:*****:*

P450-N-Z1      350 AVVGTSRLLTADDMPRLTYLRCIIDETMRLYPAAPLLLPHESSTHCKVGGY-DVPAGTML   408
P450-N-Z2      361 VSVGTSRLVTAADVPHLGYLQCIISETLRLYPAVPTLVPHESTADCVIGGHHHVPAGTML   420
                   . ******:** *:*:* **:***.**:*****.* *:****::.* :**: .*******

P450-N-Z1      409 LVNVYAIHRDPAVWDGPTEFVPERFEDGKAEGRLLMPFGMGRRKCPGETLALRTIGLVLG   468
P450-N-Z2      421 LVNGYAIHRDPATWPDPAAFRPERFEDGKAEGKFIITFGMGRRKCPGETLALRTIGLVLG   480
                   *** ********.* .*: * ***********:::: ***********************

P450-N-Z1      469 TLIQCFDWDRVDGLEVDMTESGGLTIPRAVPLEAMCRPRATMREVLQEL--           517
P450-N-Z2      481 MLIQCFDWDTADGGKVDMTEGVGITLPRAVPLEAMCRPRQTMVDVLKGLLE           531
                    ******** .** :*****. *:*:************* ** :**: *
```

Figure S2. Alignment of deduced amino acid sequences of *P450-N-Z1* and *P450-N-Z2*. Identical residues are marked by *.

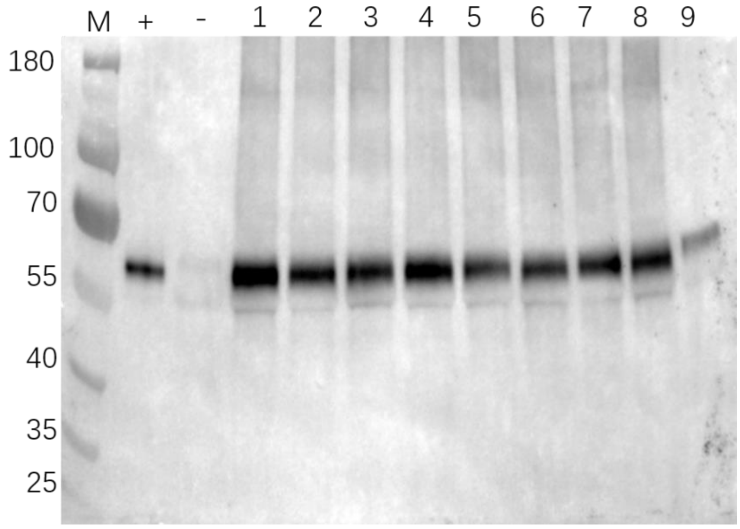

Figure S3. Western blot analysis of transgenic events of soybean at T1. M, protein molecular weight marker; +, N termins truncated (residues 4-21) P450-N-Z1 expressed in and purified from *E. coli*; -, non-transgenic plants as negative control; lanes 1 to 9, different transgenic soybean events.

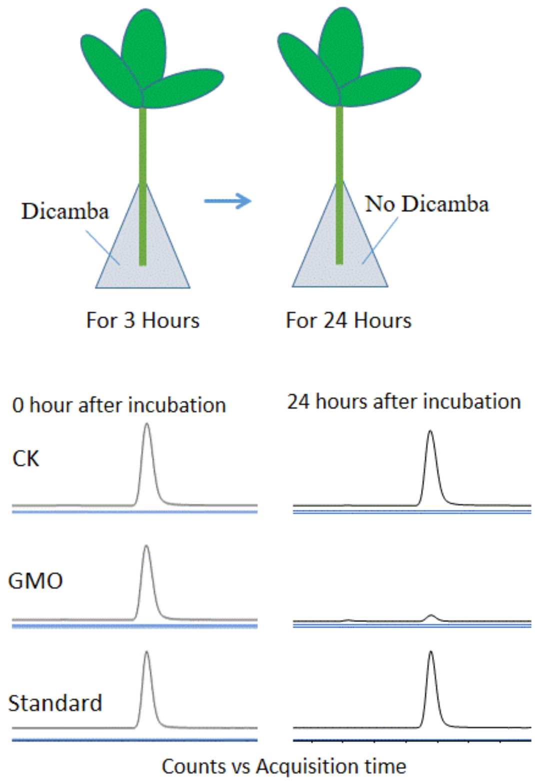

Figure S4. Dicamba was rapidly degraded by transgenic soybean expressing P450-N-Z1. Upper panel: diagram showing the soybean leaves were cut and cultured with solution containing dicamba for 3 hours, and then with solution containing no dicamba for 24 hours. Lower panel: The dicamba in the leaves were detected by HPLC chromatograms at 0 hours and 24 hours after herbicide incubation. CK, non transgenic soybean as control; GMO, transgenic soybean; Standard, chemical standard of dicamba.

Table S1. P450 genes sharing the highest sequence identity with *P450-N-Z1*.

Genbank Accession #	Species	Amino acid Sequence Identity (%) to P450-N-Z1	Herbicides tolerance activity
XP_004981686	*Setaria italica*	80	Unknow
XP_002466416	*Sorghum bicolor*	79	Unknow
XP_004981687	*Setaria italica*	79	Unknow
XP_002466414	*Sorghum bicolor*	78	Unknown
DAA51513	*Zea mays*	78	Unknown
BAO73907 (*CYP81A12*)	*Echinochloa phyllopogon*	78	Bensulfuron-methyl and penoxsulam
BAO73911 (*CYP81A22*)	*Echinochloa phyllopogon*	78	Unknown
BAO73918 (*CYP81A22*)	*Echinochloa phyllopogon*	76	Unknown
BAO73909 (*CYP81A21*)	*Echinochloa phyllopogon*	77	Bensulfuron-methyl and penoxsulam
XP_004981685.1	*Setaria italica*	77	Unknown
ACG27785.1	*Zea mays*	77	Unknown
XP_008644487 (*Nsf1*)	*Zea mays*	76	Mesotrione, sulfonylurea herbicides
ACM69387.1	*Phyllostachys praecox*	75	Unknown
BAJ94385.1	*Hordeum vulgare*	75	Unknown
XP_002466415	*Sorghum bicolor*	74	Unknown
ACG29853.1	*Zea mays*	74	Unknown
NP_001142304	*Zea mays*	74	Unknown
BAK06687.1	*Hordeum vulgare*	74	Unknown
AHZ63729.1	*Triticum aestivum*	73	Unknown
NP_001051342 (*CYP81A6*)	*Oryza sativa*	73	Bentazon and sulfonylurea herbicides
AAK38080.	*Lolium rigidum*	73	Unknown
AAK38081	*Lolium rigidum*	73	Unknown
AAK38079	*Lolium rigidum*	73	Unknown
BAD27506 (*CYP81B1v1*)	*Lolium rigidum*	73	Unknown
BAD27508 (*CYP81B1v3*)	*Lolium rigidum*	73	Unknown
BAD27507	*Lolium rigidum*	72	Unknown
BAK00283	*Hordeum vulgare*	72	Unknown
XP_003562296.2	*Brachypodium distachyon*	71	Unknown

Table S2. Expression of P450-N-Z1 in transgenic soybean lines (Mean±SD).

Event	P450-N-Z1 (μg/g fresh leaf weight)
**Event A**	19.04 (±3.17)
**Event B**	8.00 (±1.80)
**Event C**	9.04 (±1.13)
**Event D**	13.07 (±1.86)
**control**	ND

Soybean plants were planted in the greenhouse and sampled at V7 stage after a spray of flazasulfuron to separate null segregants. P450-N-Z1 was quantified by ELISA. Events A, B, C and D, transgenic soybean at T1 generation. Control, non transgenic soybean. n=3.

Table S3. P450 genes and the herbicides which they confer tolerance to.

P450s	Species	Herbicides
ADD91442 (CYP71A11)	*Nicotiana tabacum*	Chlortoluron[15]
BAH84782 (CYP81B2)	*Nicotiana tabacum*	Chlortoluron[15]
CAA78982 (CYP73A1)	*Helianthus tuberosus*	Chlortoluron[13]
O23976 (CYP76B1)	*Helianthus tuberosus*	Phenylurea herbicides: linuron, chlortoluron, isoproturon[14]
CYP76C1	*Arabidopsis thaliana*	Phenylurea herbicides: chlortoluron, isoproturon, linuron, metobromuron, metoxuron, metoxuron, monuron, diuron[16]
CYP76C2	*Arabidopsis thaliana*	Phenylurea herbicides: chlortoluron, isoproturon, linuron, metobromuron, metoxuron, metoxuron, monuron, diuron[16]
CYP76C4	*Arabidopsis thaliana*	Phenylurea herbicides: chlortoluron, isoproturon, linuron, diuron[16]
XP_003548043.3 (CYP81E22)	*Glycine max*	Bentazon[18]
AAB94584 (CYP71A10)	*Glycine max*	Phenylurea herbicides: fluometuron, linuron, chlortoluron, diuron[17]
Gh_D10G1401 (CYP749A16)	*gossypium hirsutm*	trifloxysulfuron sodium[19]
BAF97103 (CYP71C6v1)	*Triticum aestivum*	Sulfonylurea herbicides: chlorsulfuron, triasulfuron[20]
NP_001051342（CYP81A6）	*Oryza sativa*	Bentazon and sulfonylurea herbicides: MSM, TBE, BSM, PSM[21]
BAR64201.1 (CYP72A31)	*Oryza sativa*	Bispyribac sodium and bensulfuron-methyl[23]
XP_008644487 (Nsf1)	*Zea mays*	Mesotrione, bentazon, sulfonylurea herbicides[26]
BAO73907 (CYP81A12)	*Echinochloa phyllopogon*	Sulfonylurea herbicides: BSM, PX[27]
BAO73909 (CYP81A21)	*Echinochloa phyllopogon*	Sulfonylurea herbicides: BSM, PX[27]
BAF63628 (CYP71R4)	*Lolium rigidum*	Chlorotoluron[34]

QJA18355.1 (CYP81A10v7)	*Lolium rigidum*	ACCase inhibitors: diclofop-methyl, tralkoxydim; ALS inhibiting herbicide chlorsulfuron; HPPD inhibiting herbicide: mesotrione; photosystem II inhibiting herbicides: atrazine and chlorotoluron; and the tubulin inhibiting herbicide trifluralin[29]

MSM: metsulfuron-methyl; **TBE:** tribenuron-ethyl; **BSM:** bensulfuron-methyl; **PSM:** pyrazosulfuron-ethyl; **PX:** penoxsulam
